# AD risk alleles elevate microglial cGAS‐associated senescence and neurodegeneration in tauopathy

**DOI:** 10.1002/alz.084590

**Published:** 2025-01-03

**Authors:** Li Gan, Sarah Naguib

**Affiliations:** ^1^ Helen and Robert Appel Alzheimer’s Disease Research Institute, Brain and Mind Research Institute, Weill Cornell Medicine, New York, NY USA; ^2^ Weill Cornell Medicine, New York, NY USA

## Abstract

**Background:**

The strongest genetic risk factors for AD include the e4 allele of *APOE* and the *R47H* point mutation in the TREM2 receptor. TREM2 is required for the induction of a disease‐associated microglia (DAM) signature and microglial neurodegenerative phenotype (MGnD) in response to disease pathology, signatures which both include *APOE* upregulation. There is currently limited information regarding how the *TREM2‐APOE* pathway ultimately contributes to AD risk, and downstream mechanisms of this pathway are unknown. The combination of AD risk factors *R47H* and *APOE4* may help highlight the mechanisms worsening disease and contributing to increased risk in humans.

**Method:**

There are noteworthy sex differences in AD incidence and pathology, such that a greater proportion of AD patients are women, and *APOE4*‐associated risk is stronger in women. We accordingly focused our studies on female mice to examine the effects of combining three substantial risk factors: *R47H*, *APOE4*, and female sex. We chose to use the *P301S* tauopathy model for this study because tau pathology more closely aligns with cognitive decline than Ab pathology in human patients

**Result:**

Our study has found that the combination of *APOE4* and *R47H* risk factors induces neurodegeneration in female tauopathy mice without significantly impacting hippocampal tau load. A previous study similarly found that *R47H* does not affect hippocampal tau load in the *P301S* model, suggesting that *R47H* risk is driven by disease‐enhancing microglial responses to tau, rather than by tau itself. Indeed, we find that *APOE4‐R47H* exacerbates microgliosis, amplifies microglial cGAS‐STING signaling and downstream IFN response pathways in a cell‐autonomous manner, and increases cGAS‐ and BAX‐dependent microglial senescence. Our study links the strongest AD risk factors, *R47H*, *APOE4*, and female sex, with microglial cGAS‐STING activation and associated senescence in a tauopathy model, highlighting these pathways as important therapeutic targets in the treatment of AD.

**Conclusion:**

Taken together, cGAS‐STING and senescence pathways are known drivers of neurodegeneration, and our study further links upregulation of these pathways to the strongest AD risk factors. This evidence points to cGAS‐STING and senescence as vital pathological mechanisms in AD and important targets for future therapeutics.

